# Classification of postural control characteristics during quiet standing in patients with subacute stroke and their association with lesion networks

**DOI:** 10.21203/rs.3.rs-8624516/v1

**Published:** 2026-03-24

**Authors:** Shingo Hirano, Atsushi Kawaguchi, Tatsuya Igarashi, Maiko Sakamoto, Hisato Nakazono, Takanori Taniguchi, Hiroyuki Inooka, Tsubasa Mitsutake

**Affiliations:** 1 Department of Rehabilitation, Saitama Yorii Hospital, Address; 395 Youdo, Yorii-Town, Saitama 369-1201, Japan.; 2 Medical science, Saga university Graduate school, Address; 5-1-1 Nabeshima, Saga-City, Saga, 849-8501, Japan; 3 Research and Education Center for Community Medicine, Faculty of Medicine, Saga University, Address; 5-1-1 Nabeshima, Saga-City, Saga, 849-8501, Japan; 4 Department of Physical Therapy, Faculty of Health Science Technology, Bunkyo Gakuin University Address; 1196 Kamekubo, Fujimino-City, Saitama 356-0051, Japan; 5 Department of Occupational Therapy, Faculty of Medical Science, Fukuoka International University of Health and Welfare, Address; 3-6-40 Momochihama, Sawara-ku, Fukuoka 814-0001, Japan; 6 Department of Physical Therapy, Faculty of Medical Science, Fukuoka International University of Health and Welfare, Address; 3-6-40 Momochihama, Sawara-ku, Fukuoka 814-0001, Japan

**Keywords:** classification, ascending vestibular pathways, medial lemniscus, spinothalamic tract, quiet standing, structured supervised sparse principal component analysis

## Abstract

Standing balance after stroke is crucial for independence and fall prevention, yet quiet-standing postural control and its neural substrates are incompletely understood. We studied 75 patients (mean age 61.08 ± 11.76 years; 59 men) within 2 months of stroke onset. During 30-s eyes-open quiet standing, center-of-pressure (CoP) data were recorded and 52 variables were derived. We applied supervised structured sparse principal component analysis (S3PCA) to extract components and Gaussian mixture clustering to define phenotypes. Admission MRI quantified the percent damage of white-matter tracts using the Lesion Quantification Toolkit. Between-cluster differences in tract damage were assessed using Kruskal–Wallis and Dunn–Holm post hoc tests; within-cluster associations between tract damage and S3PCA scores were assessed using Spearman’s rank correlation. S3PCA yielded four components (antero-posterior frequency distribution, medio-lateral spatial, antero-posterior frequency, and medio-lateral frequency). The three following clusters emerged: (1) higher-frequency profile, (2) lower-frequency/spatial profile, and (3) low medio-lateral frequency with high spatial profile. Compared with cluster 2, cluster 3 showed greater damage in the medial lemniscus and spinothalamic tracts, and tract damage correlated positively with medio-lateral frequency scores within cluster 3. Ascending vestibular/somatosensory pathway damage is linked to phenotype-specific quiet-standing postural control after stroke.

## INTRODUCTION

Approximately half of patients with stroke experience impairments in balance, gait, and activities of daily living (ADL) [1]. Among these, standing balance is closely linked to ADL independence, the achievement of independent ambulation, and fall risk [2–4]. It is also associated with perceived difficulties in patients with stroke in everyday life [5]. Thus, standing balance affects both functional outcomes and quality of life (QOL) in this population. Accordingly, accurately characterizing postural control during quiet standing and developing interventions tailored to individual phenotypes are essential for improving ADL and QOL after stroke.

Posturography is a gold-standard method for assessing standing balance [6]. It analyzes the center of pressure (CoP) during standing and derives indices of sway area, velocity, and frequency characteristics, enabling a more comprehensive characterization of postural control than clinical outcome measures such as the Berg Balance Scale (BBS) [7]. Studies have quantified CoP during quiet standing in patients with stroke; however, findings regarding which parameters are increased relative to healthy controls have been inconsistent [8–11]. Beyond methodological differences such as stance width, sampling frequency, and computational procedures, individual differences in postural control strategies likely contribute to this heterogeneity. Notably, Fujii et al classified quiet-standing CoP characteristics into six phenotypes in Parkinson’s disease [12] demonstrating that even a single functional domain within a specific disorder can exhibit diverse pathophysiology with implications for targeted rehabilitation and clinical decision-making. Analogously, the phenotype-based classification of postural control using CoP variables in patients with stroke may delineate the heterogeneous nature of standing-balance impairment and facilitate mechanistic insights.

Postural neural control after stroke involves multiple brain regions, including the primary motor cortex, supplementary motor area, basal ganglia, and cerebellum [13], implying that balance depends not on a single locus but on distributed networks connecting multiple anatomical areas. The recently developed Lesion Quantification Toolkit (LQT) provides a structural connectomics-based approach to quantify white-matter tract damage using diffusion-weighted or conventional structural imaging [14]. Compared with diffusion tensor imaging (DTI), LQT enables comprehensive damage estimation across all major white-matter tracts and can be applied to routinely acquired magnetic resonance imaging (MRI), increasing feasibility where DTI protocols or specialized hardware are limited. Accordingly, leveraging LQT to identify white-matter pathways associated with standing-balance impairment, together with phenotype-based classifications of postural control, in patients with stroke can yield mechanistically informative and clinically actionable insights.

In this study, we aimed to exploratorily characterize postural control during quiet standing in patients with stroke and identify the white-matter pathways associated with these characteristics. Elucidating these features may provide neurologically and clinically meaningful insights into the pathophysiology of post-stroke balance impairment, thereby facilitating individualized, evidence-based decision-making and guiding the development of more effective interventions.

## METHODS

### Study designs

This single-center retrospective observational study was conducted in Japan. The study was approved by the Ethics Committee of Saitama Yorii hospital (Approval No. Y251225–1, Date: 25 December 2025) and adhered to the principles of the Declaration of Helsinki. The requirement for written informed consent was waived by the Ethics Committee of Saitama Yorii hospital because of the retrospective nature of the study, and an opt-out procedure was used. The study information was publicly posted on the hospital website, explicitly stating that de-identified individual data would be used. Patients were informed that they could opt out at any time. This study was conducted in accordance with the Strengthening the Reporting of Observational Studies in Epidemiology guidelines for observational studies [15].

### Participants

We screened 144 inpatients with a first-ever stroke admitted to our hospital between March 2022 and October 2025. Inclusion criteria were: 1) first-ever unilateral ischemic or hemorrhagic stroke; 2) ability walk independently in ADL before stroke onset; 3) ability to maintain quiet standing for 30 s without physical assistance; and 4) completion of posturography within 2 months of stroke onset. Exclusion criteria were: 1) comorbid conditions affecting balance (for example, orthopedic disorders, peripheral vestibular dysfunction); 2) severe aphasia or cognitive impairment precluding valid completion of posturography; 3) lack of MRI; or 4) refusal to participate.

### Clinical assessments

Demographic and clinical information (height, weight, age, days from onset to admission, days from onset to posturography, lesion side, and lesion location [supratentorial/infratentorial]) were extracted from electronic records. Balance ability, lower-limb motor impairment, lower-limb somatosensory function, trunk function, and ambulation were also recorded. These assessments were performed by trained physical therapists not involved in the posturography or neuroimaging analyses. All clinical assessments were completed within 1 week of the posturography session.

Balance was assessed using the BBS, a validated and reliable measure of overall balance [16], comprising 14 tasks (sit-to-stand, standing with eyes open, sitting unsupported, sitting down, transfers, standing with eyes closed, feet together, forward reach, retrieving object from the floor, turning to look behind, 360° turns, alternating foot on stool, tandem stance, and single-leg stance), each scored 0–4 for a total of 56; higher scores indicate better balance.

Lower-limb motor impairment and somatosensory function were assessed using the Fugl‒Meyer Assessment lower-extremity (FMA-LE; 0‒34) and Fugl‒Meyer Assessment sensory (FMA-S; 0‒12), respectively, both with established validity and reliability [17,18]. Higher scores reflect better function.

Trunk function was evaluated using the Trunk Impairment Scale (TIS), which provides a validated and comprehensive assessment of static and dynamic trunk control [19]. The scores range from 0–23, with higher scores indicating better function.

Ambulation was graded using the Functional Ambulation Category (FAC), which classifies walking independence from 0 (non-ambulatory) to 5 (independent outdoor ambulation) [20]. These outcome measures are the recommended clinical endpoints for stroke rehabilitation [21].

### Posturography

Posturography was performed within 1 month of admission and as early as feasible once patients could maintain quiet standing for 30 s. Participants completed a 30-s, eyes-open quiet-standing trial on a force platform (Anima, BALANCE CODER BW-6000; sampling frequency 100 Hz). A single trial was used due to clinical time constraints and fatigue considerations. Prior work has demonstrated high within-session reliability and validity of single-trial posturography in patients with stroke [22].

During testing, the upper limbs rested naturally along the body. All participants stood barefoot with their heels touching and toes abducted at 30° [23]. Participants were instructed to (1) fixate on a visual target 1.5 m ahead at eye level and (2) maintain a naturally, relaxed posture during the trial [24]. Testing was performed in a well-lit room maintained at 20–23°C. The participants wore comfortable clothing and were barefooted. Foot placement was standardized across trials using alignment marks on the platform. Throughout testing, an examiner stood within arm’s reach, without obstructing stance, to guard against falls. A support structure was also positioned within reach, but without interfering with stance, further minimizing fall risk. The positions of both examiner and support structure were standardized across all participants.

### CoP data processing

The CoP signals were low-pass filtered using a 4th-order Butterworth filter with a cut-off frequency of 10 Hz, applied bidirectionally (zero-phase). To comprehensively characterize postural control, we calculated 52 variables across four domains—static, dynamic, frequency-domain, and Stabilometric Diffusion Analysis (SDA)—as reported previously [25,26] (Table 1).

#### Spatial variables

We computed the following 11 variables: mean value in the anterio-posterior (AP) and medio-lateral (ML): MEAN AP and MEAN ML, maximal distance in AP and ML:MAX AP and MAX ML, ratio of amplitude: MAX RATIO, root mean square in AP and ML: RMS AP and RMS ML, coefficient of sway direction: COEF. SWAY DIR, 95% confidence ellipse area: 95% CONF. AREA, principal sway direction: SWAY DIR, length over area: LNA.

#### Dynamic variables

We computed 13 velocity-related measures and 4 sway density curve (SDC) related indices: mean velocity in AP and ML: MEAN VEL AP and MEAN VEL ML, velocity RMS in AP and ML: VEL RMS AP and VEL RMS ML, velocity zero-crossing in AP and ML: ZERO-CROSS AP and ZERO-CROSS ML, mean velocity peak absolute value in AP and ML: PEAK VEL AB AP and PEAK VEL AB ML, mean velocity peak positive value in AP and ML: PEAK VEL POS AP and PEAK VEL POS ML, mean velocity peak negative value in AP and ML: PEAK VEL NEG AP and PEAK VEL NEG ML, sway area per second: AREA PER SEC, mean sway density peak: PEAK SD, mean spatial distance between sway density peak: DIST. PEAK SD, mean time distance between sway density peak: TIME. PEAK SD, fractal dimension: FRAC. DIM.

#### Frequency-domain variables

We computed the following 20 variables using fast Fourier transform (FFT): total power in AP and ML: TPOWER AP and TPOWER ML, mean frequency in AP and ML: MEAN FREQ AP and MEAN FREQ ML, 50% power frequency in AP and ML: 50% POWER. F AP and 50% POWER. F ML, 95% power frequency in AP and ML: 95% POWER. F AP and 95% POWER. F ML, frequency dispersion in AP and ML: FREQ. DISP AP and FREQ. DISP ML, energy contents of <0.5 Hz in AP and ML: ENERGY <0.5 Hz AP and ENERGY <0.5 Hz ML, energy contents of 0.5‒2.0 Hz in AP and ML: ENERGY 0.5‒2.0 Hz AP and ENERGY 0.5‒2.0 Hz ML, energy contents of >2.0 Hz in AP and ML: ENERGY >2.0 Hz AP and ENERGY >2.0 Hz ML, slope of the power spectral density (PSD) for 0.15‒1.0 Hz in AP and ML: PSDL AP and PSDL ML and AP slope of the PSD for >1.0 Hz in AP and ML: PSDH AP and PSDH ML.

#### SDA

Using vector (two-dimensional) CoP coordinates, we computed the mean square displacement (MSD) as follows:

$$<\Delta x^{2}>=({\left[x(t+\Delta t)-x(t)\right]}^{2}),$$

where x denotes the CoP position vector, t is time, and Δt is the time lag. Following prior work, Δt was sampled over 0–10 s [27]. Plotting $\Delta x^{2}$ against Δt, we obtained: short-term diffusion coefficient (StD) and long-term diffusion coefficient (LtD), defined as one-half of the slope of the best-fit line in the short- and long-lag regions, respectively. The intersection of these two fitted lines yielded the critical time (CT), and the corresponding MSD value defined as the critical MSD (CMSD). CoP processing was performed using MATLAB R2024a (MathWorks, Natick, MA, USA).

### Neuroimaging analysis

Stroke diagnosis was confirmed by a physician, and imaging was processed by the investigators. For ischemic stroke, diffusion-weighted imaging (DWI; 32 slices; repetition time 3,250 ms; echo time 100.0 ms; flip angle 90°; matrix 288 × 288; field of view 230 × 230 mm) was used. For intracerebral hemorrhage, fluid-attenuated inversion recovery (FLAIR; 32 slices; repetition time 10,000 ms; echo time 119.0 ms; flip angle 90°; matrix 544 × 544; field of view 230 × 230 mm) was used. All scans were acquired within 2 months of stroke onset using a 1.5 T Vantage Elan system (Canon, Tochigi, Japan).

Preprocessing and lesion-network analyses were performed in MATLAB R2020b (MathWorks, Natick, MA, USA) using Statistical Parametric Mapping (SPM12) and the LQT [14,28,29]. First, each patient’s DWI or FLAIR image was spatially normalized to the Montreal Neurological Institute template using SPM12. Lesion maps were then created and binarized (lesion = 1; non-lesion = 0). To aggregate lesion topography, images from patients with right-hemisphere lesions were left–right flipped.

Tract-specific disconnection was estimated in the LQT by overlaying binarized lesion masks on normative structural connectivity derived from the Human Connectome Project (HCP)-842 atlas, a population-averaged template built from high-resolution DTI of 842 healthy adults [30]. We computed the proportion of damage for 40 white-matter tracts defined in the HCP-842 atlas; because of left–right flipping, when bilateral labels existed, we analyzed the left-hemisphere label only.

### Statistical analysis

#### Structured supervised sparse principal component analysis (S3PCA)

Using multiple CoP variables enables the multidimensional characterization of postural control [31]. However, a large number of variables increases model complexity and the risk of type I errors. To address this issue, we implemented S3PCA to leverage key properties of principal component analysis (PCA) while incorporating supervision, sparse, and structured constraints. Specifically, S3PCA generalizes supervised sparse PCA (S2PCA) [32] by adding a structure-inducing penalty, enabling CoP metrics to be integrated into a few coherent and interpretable factor groups aligned with the prespecified target measure. Formally, for each component score s=Xw with the data matrix X (variables × participants), the S3PCA estimates the loading vector w by maximizing

$$L(w)=(1-\mu )s^{T}s+\mu s^{T}Z-\lambda {\left\|w\right\|}_{1}\;-\;\text{Subject}\;\text{to}\;w^{T}w=1,$$

where μ controls the strength of supervision, Z is the supervisory signal (BBS), λ controls sparsity, and ${\left\|w\right\|}_{1}=\sum_{i=1}^{m}|w|$. When μ=0 and λ=0, the formulation reduces to PCA. Starting from the initialized s, we iteratively updated w using

$$w=h\lambda \left(X^{T}\{\mu s+(1-\mu )Z\},\right.$$

where $h_{\lambda }(\cdot )$ is a soft-thresholding operator. The iterations proceeded until convergence to the maximizer of L(w). For the second and subsequent components (PC2, PC3, …), variables with non-zero loadings in the preceding component were structurally zeroed, and the update was repeated; extraction stopped when all remaining loadings were zero. This procedure avoids the overlap of variables across factors and supports clearer interpretation than PCA.

These steps were followed for preprocessing. First, outliers were screened using Hotelling’s $T^{2}$ and Bonferroni adjustments (*p* < 0.05 ); and no participants were excluded. Second, following prior work, variables with Pearson correlations ≤ 0.30 or ≥ 0.90 and variables with a Kaiser–Meyer–Olkin measure of sampling adequacy ≤ 0.50were removed [12]. Seventeen variables were excluded, leaving 35 variables, which that were z-scored before analysis. We set μ=0.50, λ=0.30, and 20 iterations; the BBS served as the supervised signal Z to preserve clinical relevance. Component scores were calculated as s=Xw. All analyses were performed in R 4.3.2 (R Foundation for Statistical Computing, Vienna, Austria) using the “msma” package [32].

#### Cluster analysis with a Gaussian mixture model (GMM)

Component scores extracted by S3PCA were used for cluster analysis with the GMM, which assumes that each cluster arises from a distinct Gaussian distribution and performs a probabilistic assignment [33]. Candidate solutions with 1–75 clusters (bounded by the study sample size) were fitted. The final model was chosen based on the lowest Bayesian information criterion (BIC) and the highest integrated complete-data likelihood (ICL). All analyses were conducted in R version 4.3.2 (R Foundation for Statistical Computing, Vienna, Austria) using the mclust 5 package.

#### Between-group comparisons and correlation analyses

Baseline characteristics were compared across Gaussian-mixture clusters using the χ^2^ test (sex, lesion side, lesion location) or one-way analysis of variance, as appropriate. For all clinical outcomes and white-matter tract-specific damage proportions from the LQT, we applied the Kruskal–Wallis test across clusters. Significant pairwise differences were examined using Dunn’s post-hoc test with Holm’s correction.

Finally, we conducted an exploratory within-cluster analysis: for white-matter tracts showing significant between-cluster differences in the Dunn–Holm procedure, we assessed associations with S3PCA component scores using Spearman’s rank correlation. All analyses were performed using R version 4.3.2 (R Foundation for Statistical Computing, Vienna, Austria), with a two-sided significance level of 5%.

## RESULTS

**Figure 1:**
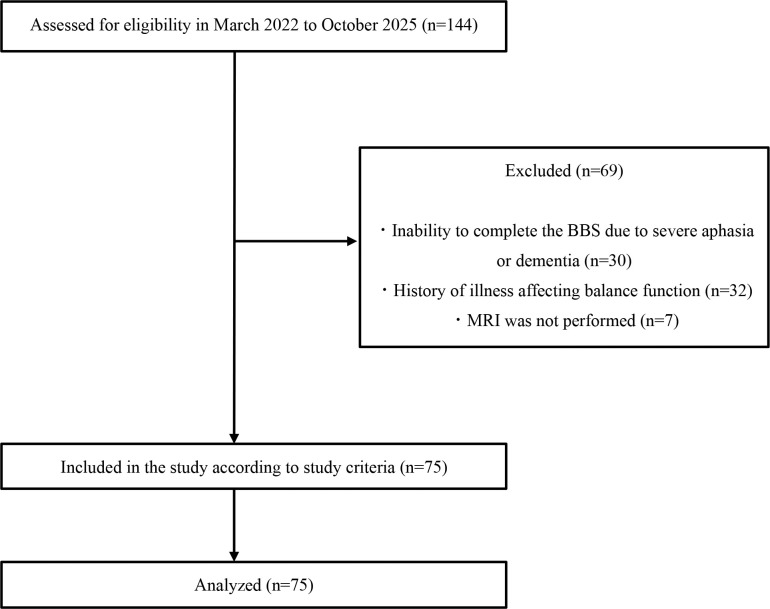
Flow chart of patients who participating in this study BBS: Berg Balance Scale, MRI: Magnetic Resonance Imaging

The participant flowchart is shown in Figure 1. Seventy-five patients were included in the final analysis. This sample size approximately satisfied an a priori power analysis (G*Power) for a one-way analysis of variance with three groups (α=0.05, 1-β=0.80, Cohen’s f = 0.40 [large], anticipated dropout rate = 10%). Table 2 summarizes the demographic and clinical characteristics. At the cohort level, median scores (range) were 46 (18–56), 32 (9–34), 12 (2–12), 18 (8–23), and 4 (1–5) for BBS, FMA-LE, FMA-S, TIS, and FAC, respectively. These results indicate mild balance and motor impairment.

### S3PCA

**Figure 2:**
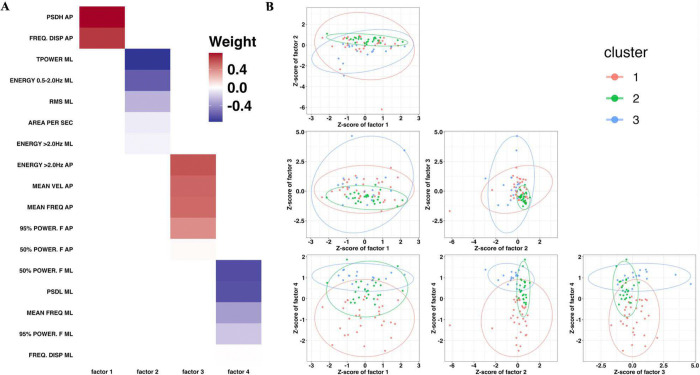
The results of structured supervised sparse principal component analysis and cluster analysis using the Gaussian mixture model **A**: The results of structured supervised sparse principal component analysis Red colors indicate a positive loading value to each factor. Blue colors indicate a negative loading value to each factor. Factor 1 represents the “antero-posterior (AP) frequency distribution component”. Factor 2 meaning “medio-lateral (ML) spatial-power component”. Factor 3 and 4 is “AP frequency components” and “ML frequency components” respectively. **B**: The results of cluster analysis using the Gaussian mixture model AP: Anterio-posterior direction, ML: Medio-lateral direction Cluster 1 showed mildly elevated scores on the antero-posterior (AP) velocity–frequency component and negative scores on the medio-lateral (ML) frequency component, indicating higher-frequency ML sway. Cluster 2 demonstrated high scores on the ML spatial component and the ML frequency component (reflecting a smaller ML sway area and lower ML sway frequency, respectively), alongside low scores on the AP velocity–frequency component. Cluster 3 exhibited low scores on the ML spatial component (indicating a larger ML sway area) and high scores on the ML and AP frequency components, consistent with lower ML sway frequency and higher AP sway frequency.

Figure 2A shows the S3PCA solution, from which the four components were extracted. Factor 1 was interpreted as an AP frequency-distribution component, driven by large positive loadings from the FREQ. DISP AP, PSDL AP. Factor 2 reflected the ML spatial component, characterized by negative loadings for RMS ML, TPOWER ML, and ENERGY 0.5–2.0Hz ML. Factor 3 represented an AP velocity–frequency component, with high positive loadings for MEAN VEL AP, MEAN FREQ AP, and 50% POWER. F AP, and 95% POWER. F AP. Factor 4 captured the ML frequency component, marked by negative loadings for MEAN FREQ ML and 50% POWER. F ML, and 95% POWER. F ML.

### Cluster analysis with a Gaussian mixture model

BIC minimization and ICL maximization identified a three-cluster solution (Figure 2B). Thirty-two participants (43.7%) were assigned to Cluster 1, 25 (33.3%) to Cluster 2, and 18 (24.0%) to Cluster 3. No significant between-cluster differences were found in demographics or clinical assessments. Regarding the S3PCA profiles, Cluster 1 exhibited slightly elevated scores for Factor 3 and negative scores for Factor 4. Cluster 2 showed higher scores for Factors 2 and 4, accompanied by negative scores for Factor 3. Conversely, Cluster 3 was characterized by negative scores on Factor 2 together with higher scores on both Factors 3 and 4.

### Between-cluster differences and correlations for white-matter tract damage.

**Figure 3:**
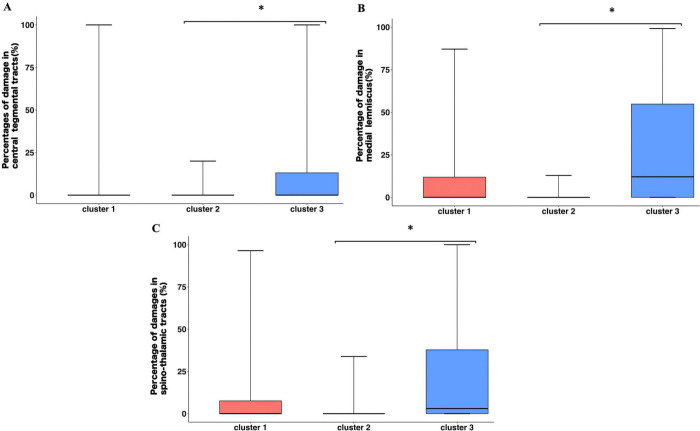
The results of Dunn’s multiple comparisons (Holm adjustment) of percentages of damage in each white-matter tract between each cluster **A:** Central tegmental tracts **B:** Medial lemniscus **C:** Spino-thalamic tracs *: p<0.05

Damage proportion in the central tegmental tract (CTT), medial lemniscus (Med.L), and spinothalamic tract (STT) revealed significant between-cluster differences in Kruskal–Wallis tests [CTT: p = 0.029, $\varepsilon^{2}=0.071$, 95%CI = 0–0.288; Med.L: p = 0.013, $\varepsilon^{2}=0.092$, 95% CI = 0.006–0.270; STT: p = 0.012, $\varepsilon^{2}=0.094$, 95% CI = 0.006–0.268, with no significant differences for the other tracts. Dunn’s tests with Holm correction showed that Cluster 3 had significantly higher damage proportions than Cluster 2 in all three tracts—the CTT (p = 0.025), Med.L (p = 0.011), and STT (p = 0.012) (Figure 3; Supplementary Tables S1 and S2). No other between-cluster differences were observed.

**Figure 4:**
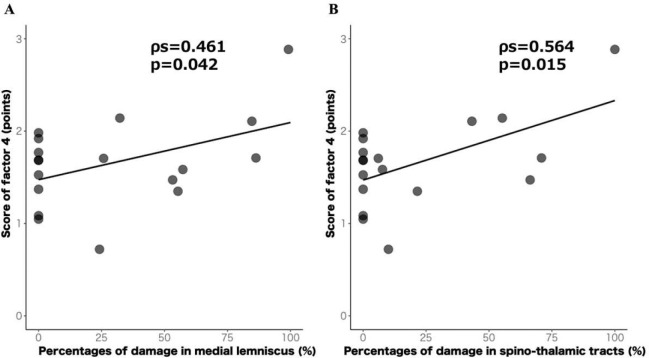
Scatter plot of the percentages of damage in each white-matter tract and score of the factor 4 in cluster 3 **A:** Medial lemniscus **B:** Spino-thalamic tracts

Figure 4 present the results of the exploratory analysis. In Cluster 3, the damage proportions in the Med.L and STT were positively correlated with the Factor 4 scores (Med.L: r = 0.461, p = 0.046, 95% CI = 0.113–0.758; STT: r = 0.564, p = 0.015, 95% CI = 0.132–0.816). No significant correlations were observed for the other tracts or clusters.

## DISCUSSION

To our knowledge, this is the first study to demonstrate that quiet-standing postural control after stroke was heterogenous rather than unitary. Using 52 CoP variables, we applied S3PCA to derive latent components and performed cluster analysis using GMM on the resulting component scores for phenotype postural control. This approach revealed three phenotypes of quiet-standing control among patients with stroke and identified lesion-network features associated with specific clusters.

Cluster analysis indicated that quiet-standing postural control in patients with stroke segregated into three phenotypes. Cluster 1 was characterized, relative to the other clusters, by slightly elevated scores on the AP velocity–frequency component and negative scores on the ML frequency component. Given that the ML frequency–defining variables loaded negatively, lower (more negative) component scores corresponded to higher ML sway frequencies. Overall, this profile suggests a control strategy dominated by high-frequency content. Elevated sway frequency has been linked to a breakdown of intermittent postural control with compensatory shifts toward more continuous control [34,35], and an elevated high-frequency CoP content signal may reflect greater voluntary, cortically mediated stabilization efforts [36]. Several frequency-based indices (MEAN FREQ AP/ML and 95% POWER. F AP/ML) and the RMS ML in Cluster 1 exceeded the mean ± 2 standard deviation values reported for healthy older adults [25], implying that these patients attempted more effortful, rapid corrective actions to counter relatively large sway during quiet standing.

Compared with the other clusters, Cluster 2 exhibited higher scores on the ML spatial and ML frequency components, indicating a smaller ML sway area and lower ML sway frequency, along with lower scores on the AP velocity–frequency component. This profile characterizes a control strategy dominated by low-frequency sway within a relatively constrained ML envelope. A reduction in dominant sway frequency has been associated with a postural regime closer to intact intermittent control [35], suggesting that patients in Cluster 2 regulate quiet standing in a manner similar to healthy patterns. However, compared with reference data from healthy older adults, the RMS ML remained approximately 1.5-fold higher, warranting caution in interpreting this cluster as “normal-like.” Notably, ML spatial variability during quiet stance has been linked to hip strategy contributions [37]. Thus, even in this relatively mildly impaired phenotype, maintaining the CoP within tight ML bounds may be challenged by biomechanical factors, particularly hip joint control, as well as sensory contributions, leading to greater lateral sway than that in healthy peers.

Compared with the other clusters, Cluster 3 showed lower scores on the ML spatial component. This finding indicates a larger ML sway area and higher scores on both ML and AP frequency components, reflecting lower dominant ML sway frequency and higher AP sway frequency. This pattern suggests a direction-specific control profile characterized by large, low-frequency ML fluctuations together with increased high-frequency AP activity. ML spatial variability during quiet stance is linked not only to hip strategy contributions [35] but also to somatosensory and vestibular inputs [38,39]. In our cohort, the degrees of lower-limb paresis, sensory deficits, and trunk control did not differ significantly across clusters. Moreover, low-frequency sway around 0.5 Hz has been associated with vestibular-driven components [40], whereas increased high-frequency AP content suggests greater voluntary, cortically mediated corrective actions and potential degradation of intermittent control [35–37]. Overall, Cluster 3 may reflect a phenotype in which the vestibular-related sway is sufficiently large to maintain the CoP within tight bounds, prompting compensatory higher-frequency AP adjustments.

Compared with Cluster 2, Cluster 3 exhibited significantly higher damage proportions in the CTT, Med.L, and STT. Within Cluster 3, damage to the Med.L and STT showed significant positive correlations with the ML frequency component scores, whereas no such associations were observed for other clusters or tracts. These findings suggest a selective link between the disruption of ascending somatosensory pathways, the dorsal column (Med.L) and spinothalamic systems, and the low-frequency sway characteristics in ML that typify Cluster 3. The CTT descends from the red nucleus through the medial pontine tegmentum to the medial medulla and terminates in the inferior olivary nucleus [41]. Lesions along this pathway can produce cerebellar ataxia and increase sway in both the ML and AP directions [42]. Moreover, postural sway characteristics in cerebellar ataxia vary substantially across individuals [43,44]. Accordingly, in Cluster 3—where a direction-specific control profile was observed, — the greater CTT damage relative to Cluster 2 may not have yielded a single robust correlation with any component, plausibly because the heterogeneity of ataxic postural control obscured a consistent linkage to a specific sway feature.

The Med.L ascends from the dorsal columns and synapse in the gracile and cuneate nuclei of the medulla; after decussation, it courses anteromedially through the brainstem to converge on the ventral posterolateral (VPL) nucleus of the thalamus [45]. This pathway conveys limb proprioceptive and cutaneous tactile inputs [45]. Adjacent to the Med.L runs the ipsilateral vestibulo-thalamic tracts (VTT), components of the ascending vestibular pathway that travel just medial and ventral to the lemniscus. Lesions here can produce vestibular symptoms such as deviations of the subjective visual vertical (SVV) [46]. Conversely, the STT originates from dorsal horn neurons, crosses within the spinal cord to the contralateral anterolateral funiculus, and ascends through the lateral medulla, lateral pontine, and midbrain tegmentum to the reticular formation and VPL nucleus, with projections to the insular and primary somatosensory cortices [47]. This system transmits crude touch/pressure and thermo-nociceptive information [47]. Notably, at the medullary level the STT courses near the vestibular nuclei, and lesions in this territory can elicit vestibular manifestations, including body lateropulsion, nystagmus, and vertigo [48,49]. As mentioned above, low-frequency sway (approximately 0.5 Hz) is linked to vestibular-driven postural sway [40]. In this study, the degree of somatosensory impairment did not differ significantly across clusters. Combined with prior evidence, this suggests that damage to the VTT running adjacent to the Med.L or involvement of the vestibular nuclei located near the STT at the medullary level may contribute to the ML frequency–driven sway characteristics observed in Cluster 3 (Supplementary Figures S1–S3). However, the FMA-S is a bedside clinical measure, and its detection threshold may be insufficient to capture subtle sensory deficits. Future studies should incorporate more sensitive somatosensory assessments, such as somatosensory-evoked potentials, to better delineate these contributions.

Clusters 1 and 2 showed no significant differences in tract-specific damage proportions, and no white-matter tract correlated significantly with any component within either cluster. Postural balance relies on distributed brain networks [13], and voluntary regulation of sway similarly recruits broad cortical territories [36]. As noted above, Cluster 1—relative to Cluster 2—exhibited increased high-frequency content associated with voluntary control and larger ML sway, and Cluster 2 also showed a larger ML sway area than the reference values in healthy older adults. These observations suggest that, particularly in patients with mildly impaired subacute stroke, sway of a certain magnitude remain amenable to voluntary regulation, and its control may depend on distributed, network-level contributions rather than on any single white-matter tract. Conversely, when sway reaches a magnitude that challenges cortical voluntary control, as in Cluster 3, impairment of the ascending vestibular pathways may play a more prominent role.

Our findings suggest that commonly used clinical scales—such as the BBS, TIS, and FAC—may be insufficient to capture phenotype-level differences in post-stroke postural control. In this cohort, none of these measures differed significantly across clusters, whereas posturography revealed distinct control profiles. Compared with bedside scales, CoP analysis provides a more sensitive and multidimensional characterization of quiet-standing balance in patients with stroke [7]. Thus, postural control in patients with stroke likely involves complex neurobehavioral features that are difficult to discern with the BBS, TIS or FAC alone. By identifying subgroups with specific control characteristics, such as larger ML sway area, lower dominant ML frequency, or greater AP high-frequency content, this approach can help identify targets for tailored rehabilitation, such as vestibular-oriented training for phenotypes with low-frequency ML sway, or strategies that modulate voluntary, cortically mediated corrections for phenotypes with prominent high-frequency components. Ultimately, a phenotype-based classification may support more precise treatment selection and monitoring than relying solely on global clinical scales.

This study had some methodological limitations. First, the retrospective design restricted the clinical battery. We did not assess muscle tone, such as Modified Ashworth Scale, or electromyography, nor did we evaluate vestibular-related indices such as SVV or nystagmus, limiting our ability to pinpoint the sensorimotor mechanisms underlying each cluster’s postural strategy. Second, our posturography protocol used a single 30-s trial. Although several trials are commonly recommended to enhance reliability [25], time constraints inherent to routine clinical care and patient fatigue precluded multiple recordings. Third, this single-center study had modest sample size, which may limit its generalizability. Future studies should address these limitations through prospective, multi-center designs, including a broader set of clinical, electrophysiological, and vestibular measures, and acquiring multiple posturography trials. Larger cohorts will allow the validation of phenotypic structure, examination of prognostic value, and testing of phenotype-specific interventions.

Despite these limitations, this is, to our knowledge, the first study to identify phenotypes of quiet-standing postural control after stroke and associations of these phenotypes with lesion-network features. These findings advance our understanding of the neural mechanisms underlying balance impairment and may facilitate more precise, phenotype-informed rehabilitation planning.

In conclusions, this study showed that quiet-standing postural control in patients with stroke can be classified into three distinct phenotypes based on CoP measurements using posturography. These phenotypic differences were not discernible using commonly employed clinical outcome scales, such as the BBS, TIS, and FAC alone. Moreover, damage proportions in the Med.L and STT were associated with sway driven by low-frequency components in the ML. Collectively, these findings enhance the pathophysiological understanding of balance impairment in patients with stroke and provide information that may facilitate phenotype-informed clinical decision-making and intervention planning.

## Supplementary Material

Supplementary Files

This is a list of supplementary files associated with this preprint. Click to download.

• Supplementaryfiles.docx

## Figures and Tables

**Table 1:** CoP parameters used in the study

Variable	Description

**Static domain**	
MEAN AP/ML	Mean position of sway (cm)
MAX AP/ML	Maximal displacement (cm)
MAX RATIO	Maximal displacement ML divided by AP (cm)
RMS AP/ML	Root mean square of displacement (cm)
COEF.SWAY DIR.	AP and ML covariates divided by RMS AP times RMS ML
95% CONF. AREA	Area of 95% confidence ellipse (cm^2^)
SWAY DIR	The angle between AP axis and the direction of the main eigenvector of PCA (°)
LNA	Total path length of CoP trajectory divided by 95% CONF. AREA
**Dynamic domain**	
MEAN VEL AP/ML	Mean velocity of CoP (cm/s)
VEL RMS AP/ML	Root mean square of CoP velocity (cm/s)
ZERO-CROSS AP/ML	Number of zero-crossing of CoP velocity (cm/s)
PEAK VEL AB AP/ML	Mean peak absolute CoP velocity (cm/s)
PEAK VEL POS AP/ML	Mean peak positive CoP velocity (cm/s)
PEAK VEL NEG AP/ML	Mean peak negative CoP velocity (cm/s)
AREA PER SEC	Triangle area formed by mean position and consecutive two time points (cm^2^)
PEAK SD	Mean peak value in sway density curve of 3mm radius (counts)
DIST. PEAK SD	Mean peak value of planar distance in sway density curve of 3mm radius (cm)
TIME. PEAK SD	Mean time interval between peak values in sway density curve of 3mm radius (s)
FRAC. DIM	Fractal dimension of CoP planar direction
**Frequency domain**	
TPOWER AP/ML	Total power value of power spectrum density (cm)
MEAN FREQ AP/ML	Mean frequency of power spectrum density (Hz)
50% POWER. F AP/ML	Median frequency of power spectrum density (Hz)
95% POWER. F AP/ML	95% percentile frequency of power spectrum density (Hz)
FREQ DESP AP/ML	Ratio of the SD of the frequency to the RMS of the frequency (Hz)
ENERGY <0.5 Hz AP/ML	Energy content below 0.5 Hz in the power spectrum density (cm)
ENERGY 0.5–2.0 Hz AP/ML	Energy content 0.5–2.0 Hz in the power spectrum density (cm)
ENERGY >2.0 Hz AP/ML	Energy content above 2.0 Hz in the power spectrum density (cm)
PSDL AP/ML	Liner regression against <1.0 Hz band in power spectrum density log plot
PSDH AP/ML	Liner regression against >1.0 Hz band in power spectrum density log plot
**SDA**	
StD	Slope of short-term range of SDA
LtD	Slope of long-term range of SDA
CT	Transition time of StD and LtD (s)
CMSD	Slope of CT

AP: Anterio-posterior direction, ML: Medio-lateral direction, CoP: Center of pressure, SDA: Stabilometric Diffusion Analysis, SD: Standard deviation

**Table 2:** Demographic and clinical characteristics

	All Patients (n=75)	Cluster 1 (n=32)	Cluster 2 (n=25)	Cluster 3 (n=18)

**Demographic characteristics**				
Age (years), mean (SD)	61.08(11.76)	62.38(12.21)	61.59(11.17)	74.00(9.26)
Height (m), mean (SD)	1.63(0.73)	1.65(0.68)	1.63(0.84)	1.60(0.55)
Weight (kg), mean (SD)	69.11(11.61)	66.66(12.48)	68.72(11.29)	58.11(11.89)
Days post-onset at the admission (days), mean (SD)	24.21(13.79)	25.00(12.43)	24.72(16.99)	21.11(11.49)
Days post-onset at the posturography (days), mean (SD)	35.48(18.07)	35.97(15.35)	35.80(23.38)	34.17(14.73)
Sex(n), (male/female)	59/16	27/5	18/7	14/4
Damage side (n), (left/right)	35/40	17/15	12/13	6/12
Damage region (n), (Supra-t, Infra-t)	51/24	19/13	21/4	11/7
**Clinical characteristics**				
BBS (points), median (range)	46.00(18.00–56.00)	46.00(20.00–56.00)	47.00(25.00–56.00)	46.00(18.00–56.00))
FAC (0/1/2/3/4/5), (n)	0/3/16/23/18/15	0/1/10/7/9/5	0/2/2/7/6/8	0/0/4/9/3/2
FMA-LE (points), median (range)	32.00(9.00–34.00)	31.00(9.00–34.00)	33.00(20.00–34.00)	30.00(14.00–34.00)
FMA-S (points), median (range)	12.00 (2.00–12.00)	12.00 (2.00–12.00)	12.00 (8.00–12.00)	12.00(4.00–12.00)
TIS (points), median (range)	18.00 (8.00–23.00)	19.00 (9.00–23.00)	17.00 (9.00–23.00)	18.00 (8.00–23.00)

SD: Standard deviation, Supra-t: Supra-tentorial stroke, Infra-t: Infra-tentorial stroke, BBS: Berg Balance Scale, FAC: Functional Ambulation Categories, FMA-LE: Fugl–meyer assessment lower extremity, FMA-S: Fugl–meyer assessment sensory, TIS: Trunk impairment scale

## Data Availability

The data that support the findings of this study are available from the corresponding author upon reasonable request.
